# A Text-Book of the Theory and Practice of Medicine

**Published:** 1893-04

**Authors:** 


A TEXT-BOOK OF THE THEORY AND PRACTICE OF MEDICINE. By American Teachers. Edited by Wm. Pepper, M. D., LL. D., Provost and Professor of the Theory and Practice of Medicine and of Clinical Medicine in the University of Pennsylvania. In two volumes. Illustrated. Philadelphia: Wm. B. Saunders, 913 Walnut Street, 1893.

The first volume of this work is now before the profession. It contains nine hundred pages of clear type. It is essentially an encyclopedia, thirteen authors having contributed their observations to make this one fine volume. Among them, Dr. J. S. Billings, the celebrated Curator of the Army Medical Museum and Library, at Washington, D. C., writes upon Hygiene, giving all the very latest ideas. Dr. William Pepper, the Provost of the University of Pennsylvania, who in addition to his very large medical practice has, as chief officer in the administration of the University, conducted that venerable institution along to such a career of prosperity that the various departments of learning into which it is divided now number nearly thirty gigantic buildings and form a very respectable village of themselves, writes upon the different fevers—ephemeral, typhoid, typhus, relapsing, cerebro-spinal, etc. He also contributes a remarkable article upon the lately prevailing influenza, known as "la grippe." It is followed by a chapter from the same pen upon Dengue. Recollecting that many physicians have claimed that grippe was only a form of dengue, the value of these two chapters, appearing at this time, and their arrangement closely together, will be understood and appreciated. Those of us who are residents of Philadelphia, and have been familiar with the singular ability of Dr. Pepper as a medical man, his powers of diagnosis amounting almost to intuition, will keenly value any of his productions, knowing that his brilliant career has ensured his writing with a gifted pen. Those who have not this knowledge of him may accept it as correct information from one who being a resident of the same city with him has had the facts frequently brought to his attention during the last twenty years. Consequently this work may be relied upon to be the best of its kind.

It will be remembered that *The American Text-Book of Surgery* was reviewed in the January number at page 62. We now suggest that for those just entering upon the study of medicine or upon its practice, and who desire to know what is the latest teaching in pathology, the two best books for them to have and diligently study would be *The American Text-Book of Surgery* and *Dr. Pepper's Text-Book of the Theory and Practice of Medicine*. Of course as homœopathists the treatment advocated would have to be passed over, and instead a resort to our materia medica should be made for the selection of the simillimum.

